# Factors Contributing to In-Hospital Mortality in Dengue: Insights from National Surveillance Data in Mexico (2020–2024)

**DOI:** 10.3390/tropicalmed9090202

**Published:** 2024-09-03

**Authors:** Eder Fernando Ríos-Bracamontes, Oliver Mendoza-Cano, Agustin Lugo-Radillo, Ana Daniela Ortega-Ramírez, Efrén Murillo-Zamora

**Affiliations:** 1 Departamento de Medicina Interna, Hospital General de Zona No. 1, Instituto Mexicano del Seguro Social, Av. Lapislázuli 250, Villa de Álvarez 28984, Mexico; 2 Facultad de Ingeniería Civil, Universidad de Colima, km. 9 Carretera Colima-Coquimatlán, Coquimatlán 28400, Mexico; 3 CONAHCyT—Facultad de Medicina y Cirugía, Universidad Autónoma Benito Juárez de Oaxaca, Ex Hacienda Aguilera S/N, Carr. a San Felipe del Agua, Oaxaca 68020, Mexico; 4 Facultad de Medicina, Universidad de Colima, Av. Universidad 333, Colima 28040, Mexico; 5 Unidad de Investigación en Epidemiología Clínica, Instituto Mexicano del Seguro Social, Av. Lapislázuli 250, Villa de Álvarez 28984, Mexico

**Keywords:** dengue, dengue virus, hospital mortality, public health

## Abstract

This study aimed to identify the factors associated with all-cause in-hospital mortality in laboratory-confirmed dengue cases from 2020 to mid-2024. A nationwide retrospective cohort study was conducted in Mexico and data from 18,436 participants were analyzed. Risk ratios (RRs) and 95% confidence intervals (CIs), estimated using generalized linear regression models, were used to evaluate the factors associated with all-cause in-hospital mortality risk. The overall case–fatality rate was 17.5 per 1000. In the multiple model, compared to dengue virus (DENV) 1 infections, DENV-2 (RR = 1.81, 95% CI 1.15–2.86) and DENV-3 (RR = 1.87, 95% CI 1.19–2.92) were associated with increased mortality risk. Patient characteristics, such as increasing age (RR = 1.02, 95% CI 1.01–1.03), type 2 diabetes mellitus (RR = 2.07, 95% CI 1.45–2.96), and chronic kidney disease (RR = 3.35, 95% CI 2.03–5.51), were also associated with an increased risk of a fatal outcome. We documented the influence of both the virus and individual susceptibility on mortality risk, underscoring the need for a comprehensive public health strategy for dengue.

## 1. Introduction

Dengue fever represents a public health challenge worldwide. The disease can manifest in a range of severities, from mild symptoms to severe and potentially fatal conditions. The factors associated with dengue fever severity and mortality risk are numerous and include host characteristics such as patient age, comorbid conditions, previous dengue virus (DENV) infections, and biochemical biomarkers [1]. In Mexico, by the end of 2022, the reemergence of DENV-3 infections was observed and differences in disease outcomes had been documented according to the identified serotype [2]. Understanding the factors contributing to mortality in dengue patients is crucial for improving clinical management and reducing death rates.

The epidemiological surveillance of dengue in Mexico is coordinated by a specialized sub-system within the National System for Epidemiological Surveillance (SINAVE). According to guidelines, patients with suspected dengue (fever and at least two of the following: nausea, rash, myalgia or arthralgia, headache or retro-ocular pain, petechiae or leukopenia) are registered in the web-based SINAVE [3]. All patients provide a venous blood sample; reverse-transcription polymerase chain reaction (RT-PCR) is performed within the first 5 days of symptom onset, and serological testing is conducted after 6 days [4]. Patients are followed until recovery or death.

This study aimed to identify the factors associated with all-cause in-hospital mortality in laboratory-positive dengue cases. By analyzing data from a national surveillance system spanning from 2020 to mid-2024, we sought to provide comprehensive insights into the epidemiological and clinical determinants of dengue-related mortality.

## 2. Materials and Methods

A nationwide retrospective cohort study was conducted in Mexico, including individuals who were hospitalized due to RT-PCR confirmed dengue. Eligible participants were those with symptom onset from 1 January 2020 to 3 June 2024 (spanning 1615 days) and were identified through the records of SINAVE [5]. Patients diagnosed through serological assays or those with missing DENV serotype data were excluded.

Demographic and clinical data, including the personal histories of non-communicable diseases, were collected from the audited surveillance system. Medical records and death certificates, when applicable, served as the primary data sources.

The main binary outcome was all-cause in-hospital mortality. We computed summary statistics, including case–fatality rate (CFR), and used generalized linear models with a binomial distribution and a log-link function to estimate risk ratios (RRs) and 95% confidence intervals (CIs) for the factors associated with an increased mortality risk. A multiple generalized linear model was constructed with dengue in-hospital fatal outcome (no/yes) as the dependent variable. The independent variables included were sex, age (years), identified DENV serotype, and preexisting comorbidities (no/yes), specifically type 2 diabetes mellitus, hypertensive disease, chronic kidney disease, and immunosuppression from any cause.

Since we analyzed publicly available anonymized data solely for academic purposes, ethics approval to conduct this research was waived.

## 3. Results

**Figure 1:**
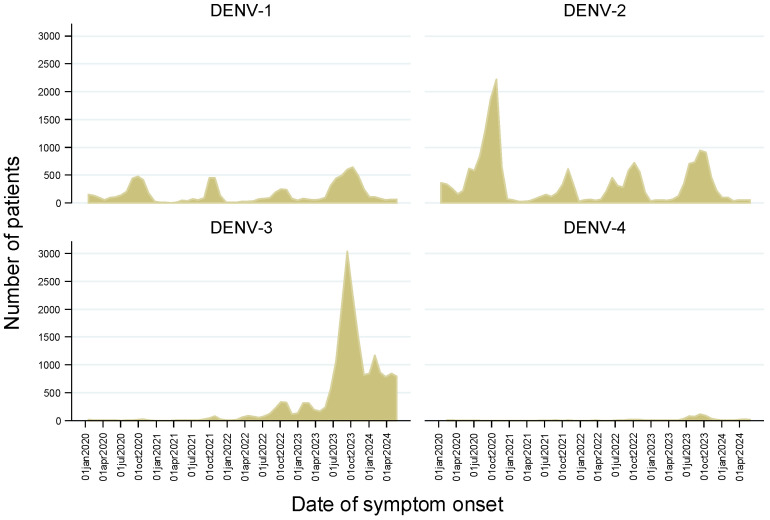
Date of symptom onset of the analyzed inpatients by identified dengue virus (DENV) serotype, Mexico 2020–2024. Note: The total number of patients by serotype was as follows: DENV-1, 2286 (12.4%); DENV-2, 7111 (38.6%); DENV-3, 8806 (47.8%); and DENV-4, 233 (1.3%).

Data from 18,436 participants were analyzed, with 323 in-hospital deaths recorded, resulting in an overall in-hospital CFR of 17.5 per 1000. The CFRs by DENV serotype were 9.2, 19.8, 17.7, and 21.5 per 1000 for serotypes 1, 2, 3, and 4, respectively. Figure 1 illustrates patient enrollment by date of symptom onset and the distribution of DENV serotypes. DENV-3 peaked from the second trimester of 2023 through to the end of the study period and was identified in 47.8% of patients.

Most participants were female (54.8%), with a mean age of 27.7 ± 17.6 years. Table 1 details the sample characteristics and in-hospital outcomes. Fatal cases were associated with older age (35.5 ± 25.4 vs. 24.5 ± 17.4 years, p < 0.001) and comorbidities such as type 2 diabetes mellitus, hypertensive disease, chronic kidney disease, and immunosuppression. The DENV serotype distribution varied significantly between fatal and non-fatal cases, with DENV-1 more common in non-fatal outcomes.

In the multiple generalized linear regression model (Table 2), DENV serotype was associated with in-hospital mortality risk. Compared to DENV-1 infection, DENV-2 was associated with an 81% increased risk (RR = 1.81, 95% CI 1.15–2.86, p = 0.010) and DENV-3 with an 87% increased risk (RR = 1.87, 95% CI 1.19–2.92, p = 0.007). The risk associated with DENV-4 was not statistically significant (RR = 1.88, 95% CI 0.73–4.86, p = 0.190).

As shown in Table 2, each additional year in age was associated with a 2% increase in risk (RR = 1.02, 95% CI 1.01–1.03, p < 0.001). Chronic preexisting comorbidities were also significantly associated with mortality risk, including type 2 diabetes mellitus (RR = 2.07, 95% CI 1.45–2.96, p < 0.001) and chronic kidney disease (RR = 3.35, 95% CI 2.03–5.51, p < 0.001).

## 4. Discussion

Our results offer insights into the epidemiology and clinical determinants of in-hospital mortality among dengue patients. These findings underscore the need for effective management strategies to mitigate the mortality rates associated with this mosquito-borne disease [6].

The association between DENV serotype and mortality risk revealed that DENV-2 and DENV-3 infections were associated with an increased mortality risk compared to DENV-1. Some published studies have documented similar findings [7,8], while others have not observed differences in disease outcomes in terms of infectious serotype [9,10], highlighting the complexity and potential influence of other factors, such as regional variations, patient demographics, and coexisting medical conditions.

The temporal distribution of DENV serotypes, particularly the peak in DENV-3 cases during 2023, underscores the dynamic nature of dengue epidemiology [11]. This temporal variation may influence disease severity and should be considered in public health interventions aimed at controlling dengue outbreaks.

We also identified demographic and clinical factors associated with mortality risk. The presence of chronic comorbidities like type 2 diabetes mellitus and chronic kidney disease further increased mortality risk, underscoring the importance of managing these underlying health conditions in dengue patients to reduce adverse outcomes. Both conditions impose a substantial burden in Mexican adults [12,13].

The potential limitations of our study must be cited. Firstly, our use of data from an epidemiological surveillance system means that our findings are contingent upon the quality of this data, which introduces the potential for inherent biases or inaccuracies. Secondly, the scope of our study is constrained by the information collected within the epidemiological surveillance system under analysis. Clinical and epidemiological data, such as prior dengue episodes, are absent. A more comprehensive analysis, incorporating data from previous dengue fever episodes, could have strengthened our manuscript. Future research addressing this gap would be valuable from both public health and clinical viewpoints.

## 5. Conclusions

These results underscore the need for a comprehensive public health strategy for dengue. We documented the influence of both the virus and individual susceptibility on disease severity. Future research should confirm these findings in diverse populations and investigate the biological pathways leading to severe dengue.

## Figures and Tables

**Table 1:** Characteristics of the study sample for selected variables and in-hospital outcomes, Mexico 2020–2024.

Characteristic	Dengue Outcome		p
	Recovery (n = 18,113)	Death (n = 323)	
Sex			
Female	9923 (54.8)	170 (52.6)	0.441
Male	8190 (45.2)	153 (47.4)	
Age (mean ± SD, years)	24.5 ± 17.4	35.5 ± 25.4	<0.001
Age group (years)			
0–9	3098 (17.1)	53 (16.4)	<0.001
10–19	6098 (33.7)	66 (20.4)	
20–29	3407 (18.8)	39 (12.1)	
30–39	2154 (11.9)	35 (10.8)	
40–49	1407 (7.8)	32 (9.9)	
50–59	962 (5.3)	32 (9.9)	
60–69	580 (3.2)	24 (7.4)	
70 or above	407 (2.3)	42 (13.0)	
Isolated serotype			
DENV-1	2265 (12.5)	21 (6.5)	0.009
DENV-2	6970 (38.5)	141 (43.7)	
DENV-3	8650 (47.8)	156 (48.3)	
DENV-4	228 (1.3)	5 (1.6)	
Type 2 diabetes mellitus			
No	17,488 (96.6)	271 (83.9)	<0.001
Yes	625 (3.4)	52 (16.1)	
Hypertensive disease			
No	17,680 (97.6)	285 (88.2)	<0.001
Yes	433 (2.4)	38 (11.8)	
Chronic kidney disease			
No	18,030 (99.5)	307 (95.1)	<0.001
Yes	83 (0.5)	16 (4.9)	
Immunosuppression (any cause)			
No	18,074 (99.8)	320 (99.1)	0.008
Yes	39 (0.2)	3 (0.9)	

Abbreviations: SD, standard deviation; DENV, dengue virus. Notes: The *p*-value from ji-squared tests is presented, except if the mean and SD is specified, and there the *t*-test was used.

**Table 2:** Factors associated with all-cause in-hospital mortality risk for dengue, Mexico 2020–2024.

	RR (95% CI), p	
	Bivariate Analysis	Multiple Analysis
Sex		
Female	1.00	1.00
Male	1.09 (0.88–1.35), 0.441	1.12 (0.90–1.39), 0.300
Age (years)	1.03 (1.02–1.04), <0.001	1.02 (1.01–1.03), <0.001
Isolated serotype		
DENV-1	1.00	1.00
DENV-2	2.16 (1.37–3.41), 0.001	1.81 (1.15–2.86), 0.010
DENV-3	1.93 (1.23–3.03), 0.005	1.87 (1.19–2.92), 0.007
DENV-4	2.34 (0.89–6.14), 0.085	1.88 (0.73–4.86), 0.190
Type 2 diabetes mellitus		
No	1.00	1.00
Yes	5.03 (3.78–6.70), <0.001	2.07 (1.45–2.96), <0.001
Hypertensive disease		
No	1.00	1.00
Yes	5.09 (3.67–7.04), <0.001	1.49 (0.99–2.23), 0.054
Chronic kidney disease		
No	1.00	1.00
Yes	9.65 (6.08–15.32), <0.001	3.35 (2.03–5.51), <0.001
Immunosuppression (any cause)		
No	1.00	1.00
Yes	4.11(1.37–12.28), 0.012	2.86 (0.99–8.29), 0.053

Abbreviations: RR, risk ratio; CI, confidence interval; DENV, dengue virus. Notes: (1) Log-binominal generalized linear regression models were employed to obtain RRs and 95% CIs; (2) Estimates from the multiple model were adjusted by all the variables listed in the table.

## Data Availability

The data presented in this study are publicly available and were provided by the General Directorate of Epidemiology of the Government of Mexico, which updates them on a weekly basis. The data can be accessed at the following link: https://www.gob.mx/salud/documentos/datos-abiertos-bases-historicas-de-enfermedades-transmitidas-por-vector (accessed on 21 July 2024).
